# Pathway Signature and Cellular Differentiation in Clear Cell Renal Cell Carcinoma

**DOI:** 10.1371/journal.pone.0010696

**Published:** 2010-05-18

**Authors:** Han W. Tun, Laura A. Marlow, Christina A. von Roemeling, Simon J. Cooper, Pamela Kreinest, Kevin Wu, Bruce A. Luxon, Mala Sinha, Panos Z. Anastasiadis, John A. Copland

**Affiliations:** 1 Department of Hematology/Oncology, Mayo Clinic, Jacksonville, Florida, United States of America; 2 Department of Cancer Biology, Mayo Clinic, Jacksonville, Florida, United States of America; 3 Department of Pathology, Mayo Clinic, Jacksonville, Florida, United States of America; 4 Institute for Translational Science and Department of Biochemistry and Molecular Biology, University of Texas Medical Branch, Galveston, Texas, United States of America; Baylor College of Medicine, United States of America

## Abstract

**Background:**

Clear cell renal cell carcinoma (ccRCC) is the most common kidney cancer. The purpose of this study is to define a biological pathway signature and a cellular differentiation program in ccRCC.

**Methodology:**

We performed gene expression profiling of early-stage ccRCC and patient-matched normal renal tissue using Affymetrix HG-U133a and HG-U133b GeneChips combined with a comprehensive bioinformatic analyses, including pathway analysis. The results were validated by real time PCR and IHC on two independent sample sets. Cellular differentiation experiments were performed on ccRCC cell lines and their matched normal renal epithelial cells, in differentiation media, to determine their mesenchymal differentiation potential.

**Principal Findings:**

We identified a unique pathway signature with three major biological alterations—loss of normal renal function, down-regulated metabolism, and immune activation–which revealed an adipogenic gene expression signature linked to the hallmark lipid-laden clear cell morphology of ccRCC. Culturing normal renal and ccRCC cells in differentiation media showed that only ccRCC cells were induced to undergo adipogenic and, surprisingly, osteogenic differentiation. A gene expression signature consistent with epithelial mesenchymal transition (EMT) was identified for ccRCC. We revealed significant down-regulation of four developmental transcription factors (GATA3, TFCP2L1, TFAP2B, DMRT2) that are important for normal renal development.

**Conclusions:**

ccRCC is characterized by a lack of epithelial differentiation, mesenchymal/adipogenic transdifferentiation, and pluripotent mesenchymal stem cell-like differentiation capacity *in vitro*. We suggest that down-regulation of developmental transcription factors may mediate the aberrant differentiation in ccRCC. We propose a model in which normal renal epithelial cells undergo dedifferentiation, EMT, and adipogenic transdifferentiation, resulting in ccRCC. Because ccRCC cells grown in adipogenic media regain the characteristic ccRCC phenotype, we have indentified a new *in vitro* ccRCC cell model more resembling ccRCC tumor morphology.

## Introduction

Renal cell carcinoma is the eighth most common cancer and highly lethal, accounting for about 13,000 deaths in the U.S. in 2009 [1]. If detected in early stages, it is potentially curable by surgical resection; however, there is no curative treatment for metastatic RCC. A better understanding of RCC's biology is essential to improving prognosis.

ccRCC represents the most common subtype (83%) of RCC [2]. The cell of origin and cellular differentiation program in ccRCC is not definitively known but ccRCC is commonly thought to arise from renal proximal epithelial cells [3]. The most striking phenotypic feature of ccRCC is its clear cell morphology, which has been linked to lipid and glycogen accumulation [4]. Thus the mechanism behind clear cell morphology is an important component of renal carcinogenesis that has yet to be defined at the molecular level.

The gene expression profile of ccRCC has been investigated at the single gene level in multiple studies [5];[6] that have compared ccRCC to normal renal samples and other histological subtypes of RCC. Many statistically significant individual genes have been identified. At present, no comprehensive analysis of ccRCC at the pathway level exists. Since biological processes are mediated by multiple genes working in synchrony and genes are co-regulated, pathway analysis is more conclusive regarding biologic changes. Notably, genes that don't seem differentially expressed at a statistically significant level may reveal biologically significant changes when studied in ontology groups [7]. Moreover, a comparison between tumor and normal tissue generates a high number of differentially expressed genes, which makes biological interpretation difficult. As a result, we conclude it is important to study gene expression profiles comprehensively by grouping individual genes into gene sets or pathways to extract more sophisticated biological interpretations.

We used SigPathway [8], a genome-wide biological pathway analysis package, and discovered three major biologic alterations in early-stage ccRCC that are likely related to a loss of epithelial differentiation. Our findings revealed four known renal developmental transcription factors (DTFs) that were significantly down-regulated in ccRCC and likely related to the differentiation changes in renal carcinogenesis. Further analysis revealed a novel molecular signature consistent with adipogenic transdifferentiation. This adipogenic molecular signature can explain the “clear cell” RCC morphology noted by cytoplasmic lipid accumulation. We also revealed a gene expression signature consistent with EMT in ccRCC. Based on these findings, we propose a carcinogenic cellular differentiation model of normal renal epithelial cells (NREs) that includes dedifferentiation, EMT, and adipogenic transdifferentiation that results in ccRCC.

## Results

### Pathway Signature of ccRCC with three major biological alterations

SigPathway analysis of our gene expression data was performed to identify a pathway signature of ccRCC. The top 40 differentially expressed pathways in ccRCC are listed in **Table 1**. Three biological themes are identified in the pathway signature of ccRCC, that include (1) loss of normal renal function, (2) down-regulation of metabolism, and (3) activated immune pathways.

**Table 1:** Pathway signature of clear cell renal cell carcinoma.

Overall Rank	Gene Set Category	Pathway	Set Size	% Up	NTk Rank	NEk Rank
		**Pathways related to normal renal function**				
1	GO:0007588	excretion	40	2	3	4
7	GO:0006814	sodium ion transport	59	12	10	15
8	GO:0031402	sodium ion binding	53	11	12	21
28	GO:0006821	chloride transport	25	20	104	5
40	GO:0005249	voltage-gated potassium channel activity	38	42	354	24
22	GO:0006818	hydrogen transport	56	7	18	69
26	GO:0015992	proton transport	55	7	21	79
		**Metabolic pathways**				
16	GO:0051258	protein polymerization	33	79	56	8
27	GO:0016830	carbon-carbon lyase activity	28	29	102	1
		**Lipid metabolic pathways**				
2	KEGG	Butanoate_metabolism	49	2	1	10
4	KEGG	Propanoate_metabolism	42	5	6	16
15	KEGG	Fatty_acid_metabolism	69	6	11	53
32	KEGG	Benzoate_degradation_via_CoA_ligation	63	30	122	7
		**Energy metabolic pathways**				
5	GO:0016614	oxidoreductase activity, acting on CH-OH group of donors	83	16	9	13
6	GO:0016616	oxidoreductase activity, acting on the CH-CH group of donors, NAD or NADP as acceptor	75	13	8	17
10	GO:0045333	cellular respiration	34	6	19	25
12	GO:0016903	oxidoreductase activity, acting on the aldehyde or oxo group of donors	31	19	50	2
18	GO:0006752	group transfer coenzyme metabolism	59	8	15	56
21	GO:0006119	oxidative phosphorylation	72	1	2	77
23	GO:0016627	oxidoreductase activity, acting on the CH-CH group of donors	34	21	68	19
25	KEGG	ATP_synthesis	44	5	23	73
29	GO:0006753	nucleoside phosphate metabolism	43	2	25	96
31	GO:0005743	mitochondrial inner membrane	70	7	14	109
		**Protein and amino acid metabolic pathways**				
3	KEGG	Valine,_leucine_and_isoleucine_degradation	55	7	4	9
24	BioCyc	valine degradation I	21	14	86	3
33	GO:0044270	nitrogen compound catabolism	55	11	16	122
34	GO:0009310	amine catabolism	53	11	24	136
35	GO:0009063	amino acid catabolism	52	10	22	139
37	GO:0008652	amino acid biosynthesis	34	38	247	12
		**Carbohydrate metabolic pathways**				
36	KEGG	Fructose_and_mannose_metabolism	48	38	187.5	6
38	KEGG	Pentose_phosphate_pathway	25	40	247	22
		**Immune pathways**				
9	GO:0009615	response to virus	43	86	17	18
11	GO:0030333	antigen processing	42	98	5	44
13	GO:0019882	antigen presentation	46	89	7	50
14	GO:0019883	antigen presentation, endogenous antigen	25	100	13	47
17	GO:0050865	regulation of cell activation	22	86	48	20
19	GO:0019885	antigen processing, endogenous antigen via MHC class I	25	100	20	52
20	GO:0051249	regulation of lymphocyte activation	20	85	64	14
		**Other pathways**				
30	GO:0003690	double-stranded DNA binding	25	76	110	11
39	GO:0003777	microtubule motor activity	33	39	316	23

Top differentially expressed pathways between early-stage ccRCC and matched normal renal tissues by SigPathway are shown. The pathway signature is characterized by three major biological alterations: downregulation of pathways related to normal renal function, downregulation of metabolic pathways, and immune pathway activation. NTK is a measure of the degree to which a given pathway differs from the other pathways. NEK is a measure of the degree to which the pathway composite expression differs among phenotypes.

**Figure 1:**
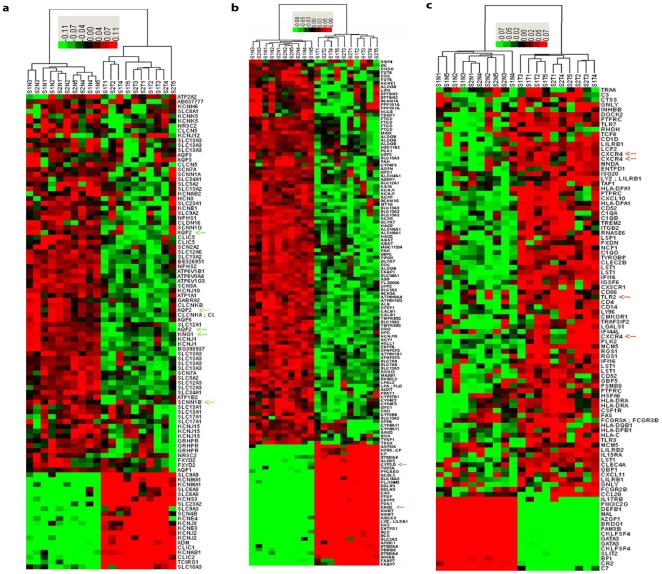
Heatmap signature of clear cell renal cell carcinoma. **a.** Normal renal function-related pathway gene expression signature. **b.** Metabolic pathway gene expression signature. **c.** Immune pathway gene expression signature. Upregulation of genes is indicated in red, downregulation is indicated in green, and a similar expression is indicated in black, as generated by Cluster 3.0.

All pathways related to normal renal function are down-regulated and include excretion, sodium ion transport, sodium ion binding, hydrogen transport, proton transport, chloride transport, and voltage-gated potassium channel activity. The excretion pathway is the most significant differentially expressed pathway in ccRCC in terms of overall rank, indicating that loss of normal renal function is the most striking biologic alteration. Other identified pathways are related to ion and electrolyte transport. Our heatmap presents the most noteworthy differentially expressed genes from these pathways (**Fig. 1a**).

The metabolic pathways are predominantly down-regulated and can be classified into 4 groups. (1) The lipid metabolic pathways are at the top of the list and include butanoate metabolism, propanoate metabolism, fatty acid metabolism and benzoate degradation via CoA ligation. (2) Energy metabolic pathways include oxidoreductase activity which acts on CH-OH group of donors, oxidoreductase activity which acts on the CH-OH group of donors, NAD or NADP as acceptor, cellular respiration, oxidoreductase activity, acting on the aldehyde or oxo group of donors, group transfer coenzyme metabolism, oxidative phosphorylation, oxidoreductase activity which acts on the CH-CH group of donors, ATP synthesis, nucleoside phosphate metabolism, and mitochondrial inner membrane. (3) Protein and amino acid metabolic pathways include valine, leucine, and isoleucine degradation, valine degradation, nitrogen compound catabolism, amine catabolism, amino acid catabolism and amino acid biosynthesis. (4) Carbohydrate metabolic pathways include fructose and mannose metabolism and pentose phosphate pathway. We have included the most differentially expressed metabolic genes from these pathways in a heatmap (**Fig. 1b**).

The immune pathways are significantly upregulated and include response to virus, antigen processing, antigen presentation, antigen presentation of endogenous antigen, regulation of cell activation, antigen processing related to endogenous antigen via MHC class I, and the regulation of lymphocyte activation. We present a heatmap generated by cluster analysis of the immune genes with significant alterations (**Fig. 1c**).

### Validation studies

**Figure 2:**
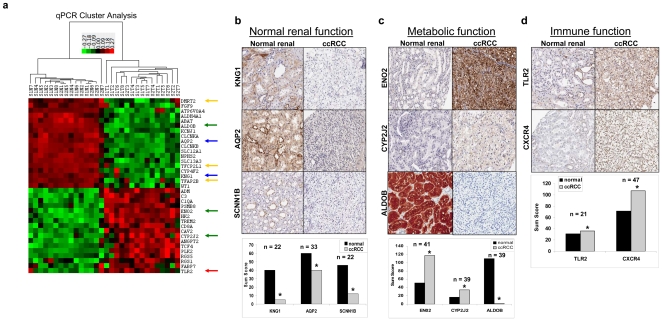
Validation of gene expression findings in clear cell renal cell carcinoma. **a.** Heatmap generated with qPCR data of DTFs and various genes related to three biological alterations. Upregulation of genes is indicated in red, downregulation is indicated in green, and similar expression is indicated in black, as generated by Cluster 3.0. **b.** IHC of proteins related to normal renal function (KNG1, AQP2, SCNN1B). **c.** Immune function (TLR2, CXCR4). **d.** Metabolic function (ENO2, CYP2J2,ALDOB). This pattern of expression is in accord with the microarray findings. Sum scores are shown with *n* as indicated. *p<0.01 when comparing ccRCC to normal match.

Validation at messenger RNA levels was performed by qPCR on several selected genes from the three pathway categories (**Fig. 2a**) and is consistent with the microarray data. IHC validation of the most significant genes in the three pathway categories was consistent with microarray and qPCR data, compared to patient-matched renal tissues samples (**Fig. 2b, 2c, 2d**). This includes the decreased expression of kininogen 1 (KNG1, NM_000893), aquaporin 2 (AQP2, AW015506), and sodium channel nonvoltage-gated 1β (SCNN1B, NM_000336) in normal renal function (blue arrows: **Fig. 2a and 2b**). Increased expression of enolase γ (ENO2, NM_001975) and cytochrome P450-family 2-subfamily J-polypeptide 2 (CYP2J2, NM_000775), and decreased expression of Fructose-1,6-bisphosphate aldolase (ALDOB, BF195998) were identified as part of the metabolic alterations (green arrows: **Fig. 2a and 2c**). Increased expression was observed in toll-like receptor 2 (TLR2, NM_003264) and C-X-C chemokine receptor type 4 (CXCR4, AJ224869), which relates to immune function (red arrows: **Fig. 2a and 2d**). Validations of altered expressions of KNG1, SCNN1B, ALDOB, and TLR2 are novel and are reported for the first time in ccRCC.

### ccRCC is characterized by a loss of renal developmental transcription factors

**Figure 3:**
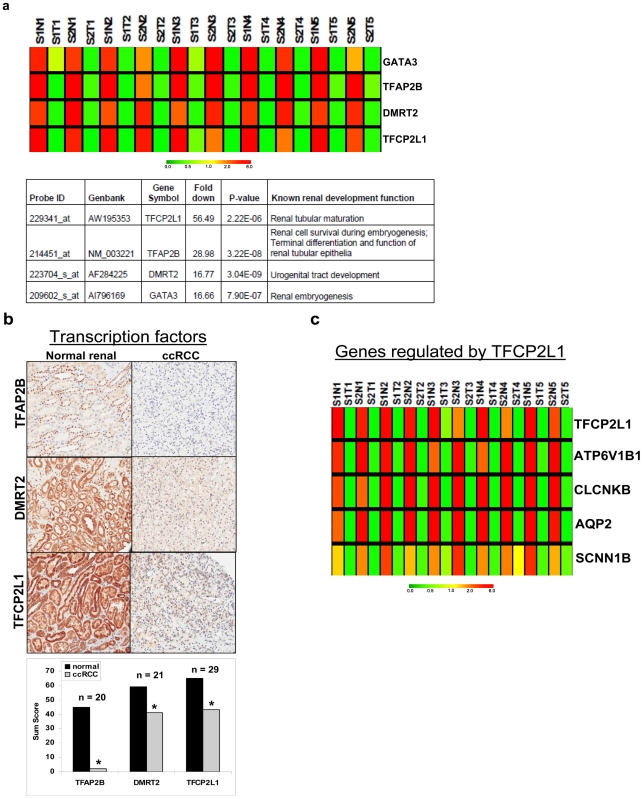
Loss of developmental transcription factors in clear cell renal cell carcinoma. **a.** A microarray heatmap showing significant downregulation of four developmental transcriptional factors in ccRCC and a table showing fold changes of DTFs and their known renal developmental function. **b.** IHC validation of decreased expression of TFAP2B, DMRT2, and TFCP2L1. Sum scores are shown with *n*, as indicated. *p<0.01 when comparing ccRCC to normal match. **c.** Microarray heatmap showing downregulation of TFCP2L1 and its regulated genes in ccRCC. S–Stage, N–normal, T–tumor. Upregulation of genes is indicated in red, downregulation is indicated in green, and similar expression is indicated in yellow, as generated by Genetree.

The pathway signature showed that ccRCC cells are quite different from normal renal cells, the bulk of which are epithelial cells. This indicates a loss of epithelial differentiation in ccRCC because the top-rated biological alteration is a down-regulation of pathways that are related to normal renal epithelial function. A lack of tubular architecture in ccRCC histology also suggests a loss of epithelial differentiation. We performed a comprehensive analysis to identify significant gene alterations in transcription factors that are known to be important for kidney development and cellular differentiation. We first identified all transcriptional factors in our dataset by using Gene Ontology gene set on transcription (GO: 006350). Statistical analysis with ANOVA was performed to derive a list of significantly altered transcription factors in ccRCC compared to normal renal samples. We then queried the literature for those transcription factors known to regulate renal development and differentiation. Four renal developmental transcription factors—GATA binding protein 3 (GATA3, AI796169), Transcription factor CP2-like 1 (TFCP2L1, AW195353), transcription factor activating protein -2β (TFAP2B, NM_003221) and Doublesex - mab-3 related transcription factor 2 (DMRT2, AF284225)—were significantly down-regulated in ccRCC (**Fig. S1**) and validated in another independent data set by qPCR consisting of 15 patient-matched normal renal and ccRCC tissues (**Fig. 2a**: yellow arrows). This data is illustrated by a heatmap and a table that outlines these transcription factors' roles in renal development and show statistically significant P values for down-regulation in ccRCC (**Fig. 3a**). Decreased expression of TFCP2L1, TFAP2B, and DMRT2 were also validated by IHC (**Fig. 3b**). Decreased GATA3 mRNA and protein expression has also been confirmed in a recent publication from our laboratory [9].

TFCP2L1 was down-regulated 56-fold and was the most down-regulated transcription factor in ccRCC. Using genes known to be regulated by TFCP2L1 from another study that uses a transgenic mouse model [10], we identified TFCP2L1-regulated genes in our microarray dataset (**Fig. 3c**). These genes, which mediate epithelial cell development and tubule formation, were present in the normal renal function-related pathways and were down-regulated in ccRCC tissues. It is likely that a loss of TFCP2L1 in ccRCC plays a role in the loss of epithelial differentiation and function in ccRCC.

### ccRCC is characterized by adipogenic transdifferentiation

Since kidneys are mesenchymal in embryonic origin and ccRCC tissues express mesenchymal markers such as vimentin and fibronectin [11], we hypothesized that ccRCC cells have undergone an epithelial to mesenchymal transition (EMT). Because we know that ccRCC cells are ladened with lipids [4] and that they histologically resemble adipocytes, we further hypothesized that our ccRCC cells may have undergone adipogenic transdifferentiation, a type of mesenchymal differentiation.

**Figure 4:**
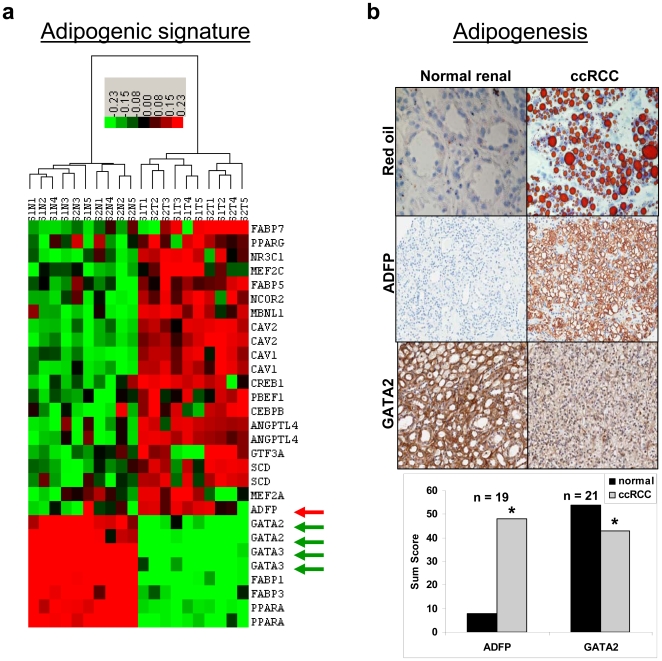
Adipogenic differentiation signature in clear cell renal cell carcinoma. **a.** Heatmap showing adipogenic gene expression signature in ccRCC. Upregulation of genes is indicated in red, downregulation is indicated in green, and similar expression is indicated in black. **b.** IHC showing lipid-laden clear cell morphology of ccRCC, increased expression of ADFP, and decreased expression of GATA2 in ccRCC. Sum scores are shown with *n*, as indicated. *p<0.01 when comparing ccRCC to normal match.

We confirmed lipid accumulation by IHC using oil Red ‘O’ stain (**Fig. 4b**) and we identified a gene expression signature in ccRCC that is consistent with adipogenic differentiation (**Fig. 4a**). The 22 genes used for the hierarchical cluster analysis came from the adipogenesis human pathway [12] and literature review [13]. Peroxisome proliferator-activated receptor γ (PPARγ, NM_015869) and CCAAT/enhancer binding protein β (CEBPβ, AL564683) are transcription factors known to be required for adipogenesis and are upregulated in ccRCC. PPARα (BC004162), known to promote lipid degradation and β-oxidation of fatty acids, was downregulated [14];[15];[16]. Adipose differentiation-related protein (ADFP, BC005127), which is a marker of adipocyte differentiation and lipid storage [5], was significantly upregulated and validated by IHC (**Fig. 4b**). This upregulation of ADFP at the mRNA and protein level in ccRCC cells, is consistent with published data [5]. Angiopoietin-like 4 (ANGPTL4, NM_016109), which increases triglycerides by potent inhibition of lipoprotein lipase [17], was increased in ccRCC tumor tissue by 23-fold and may play a major role in lipid accumulation. Further, ANGPTL4 is upregulated during adipocyte differentiation [18]. Stearoyl-CoA desaturase (SCD, AB032261), which is involved in lipogenesis [19], was also upregulated. GATA2 (AL563460) and GATA3, both known to inhibit adipocyte differentiation [20];[21], were down-regulated and both were validated by IHC (**Fig. 4b** and [9]). Caveolin-1 (CAV1, NM_001753), which has been linked to lipid droplet biology and neutral lipid storage [22];[23], was significantly upregulated. Fatty acid binding proteins (FABPs) are significantly altered in their expression in ccRCC; FABP5 & 7 (NM_001444 & NM_001446) were upregulated, while FABP1 & 3 (NM_001443 & AI041520) were down-regulated. This pattern of directional alterations of adipogenic gene expression in ccRCC is consistent with adipogenic transdifferentiation. We further validated our findings by comparing data from a publicly available gene expression database (GSE#15641, deposited at Gene Expression Omnibus) of early-stage ccRCC against normal renal samples (**Fig. S2**). Thus, we have discovered a novel adipogenic signature in ccRCC.

### ccRCC cells undergo induced adipogenic and osteogenic transdifferentiation

**Figure 5:**
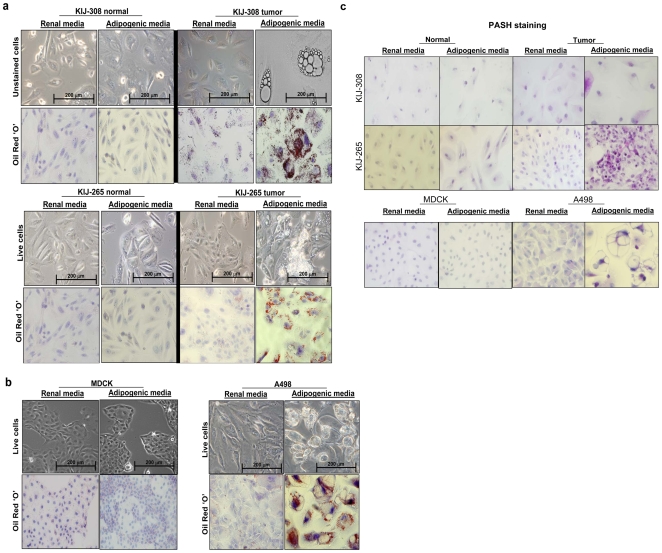
Adipogenic differentation in clear cell renal cell carcinoma. **a.** Cellular differentiation experiments showing that KIJ-308 and KIJ-265 ccRCC cells are capable of adipogenic differentiation and become lipid-laden in adipogenic media, as indicated by Oil Red ‘O’ staining. Normal patient-matched cells were unable to differentiate. **b.** A498 ccRCC cells are also capable of adipogenic differentiation, as indicated by Oil Red ‘O’ staining, unlike normal renal canine MDCK cells. **c.** Under adipogenic media conditions, ccRCC also produces glycogen, as shown by a PASH stain.

**Figure 6:**
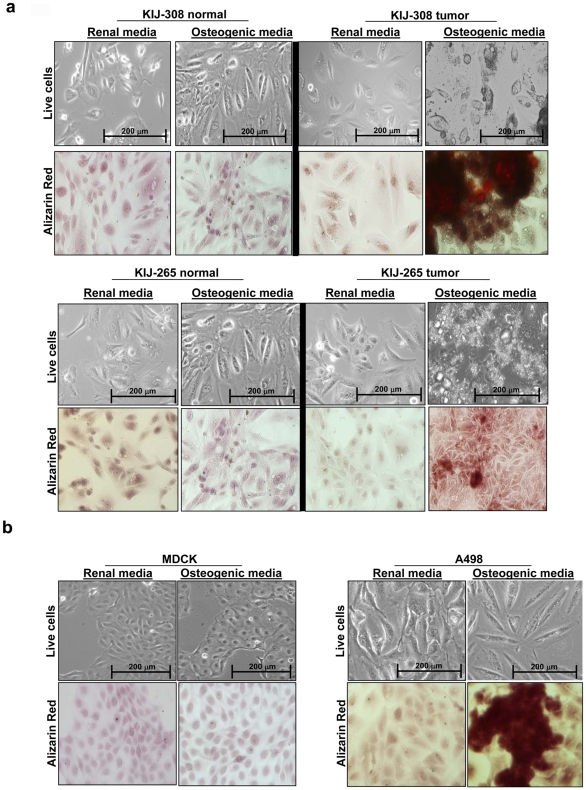
Osteogenic differentation in clear cell renal cell carcinoma. **a.** Cellular differentiation experiments showing that KIJ-308 and KIJ-265 ccRCC cells are capable of osteogenic differentiation in osteogenic media by developing calcium deposits, as shown by Alizarin Red stain. Normal patient-matched cells were unable to differentiate. **b.** A498 ccRCC cells are also capable of osteogenic differentiation, as shown by Alizarin Red stain, unlike normal renal canine MDCK cells.

Using our newly discovered adipogenic ccRCC signature, we performed differentiation experiments on three human ccRCC cell lines to test the adipogenic differentiation capacity of ccRCC in adipogenic media. All ccRCC tumor cells grown in adipogenic media underwent adipogenic transdifferentiation as revealed by oil red ‘O’ staining for lipid droplets (**Fig. 5a, 5b**). Glycogen accumulation was also observed through the use of Periodic Acid Schiff-Hematoxylin (PASH) stain in adipogenic ccRCC tumor cells (**Fig. 5c**). This transdifferentiation is unique to ccRCC because adipogenic changes were not seen in controls (tumor cells in renal media, MDCK and patient-matched normal renal epithelial cells in both medias, **Fig. 5**). After this discovery, we performed osteogenic transdifferentiation experiments on the four cell lines to determine whether or not ccRCC cells were capable of other types of mesenchymal differentiation. Surprisingly, osteogenic differentiation, as verified by Alizarin stain, was observed in ccRCC cells grown in osteogenic media, but not in any of the controls **(** **Fig. 6**).

### ccRCC is characterized by EMT molecular markers

**Figure 7:**
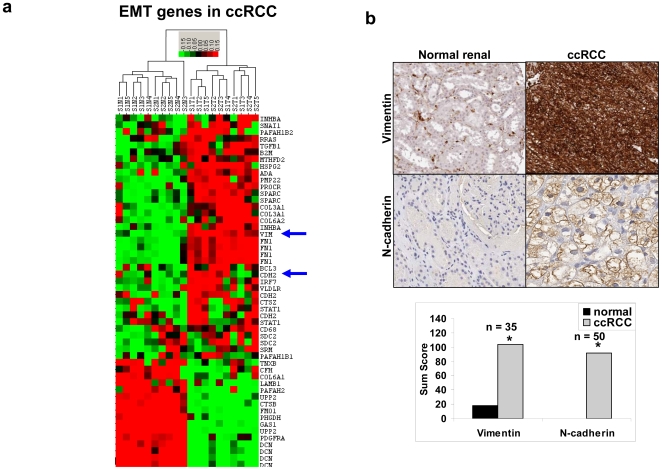
Epithelial mesenchymal transition in ccRCC. **a.** A heatmap showing the increased expression of some markers associated with EMT. Upregulation of genes is indicated in red, downregulation is indicated in green, and similar expression is indicated in black. **b.** IHC validation of two known markers of EMT: vimentin and N-cadherin. Sum scores are shown with *n*, as indicated. *p<0.01 when comparing ccRCC to normal match.

Genomic profiling identified known molecular markers of EMT [24]. Utilizing data bases and the literature searches we compiled a list of genes known to be involved in EMT [24] and selected 40 EMT genes from our ccRCC data set that represented an EMT genotype (**Fig. 7a**). Well known upregulated molecular markers include N-cadherin (CDH2, M34064), fibronectin (FN1, AK026737), transforming growth factor β1 (TGFβ1, BC000125) and vimentin (VIM, A1922599) (**Fig. 7a**). We validated vimentin (VIM) and N-cadherin (CDH2) at the protein level (**Fig. 7b**). Vimentin protein expression in ccRCC has been previously described by us and others [25], [26]. Our results agree with previously reported findings that reveal upregulated expression of fibronectin in ccRCC at the gene and protein levels [11], [27], [28]. In summary, the pattern of directional alterations in our gene set indicates for an EMT gene expression signature and is consistent with an EMT phenotype. We further validated our findings by comparing a publicly available gene expression database (GSE#15641) of early-stage ccRCC against normal renal samples (**Fig. S3**).

### Cellular differentiation model in pathogenesis of ccRCC

**Figure 8:**
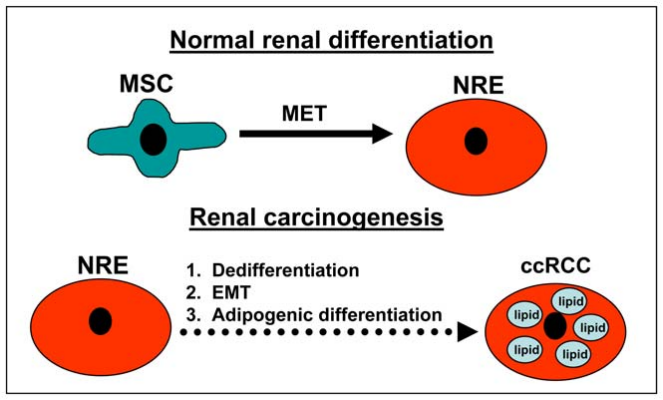
Proposed model for cellular differentiation in ccRCC. During normal renal development, mesenchymal stem cells undergo mesenchymal epithelial transition (MET) to develop into normal renal epithelial cells (NREs). In renal carcinogenesis, NREs undergo de-differentiation and epithelial mesenchymal transition (EMT), followed by adipogenic differentiation to develop into ccRCC.

Based on our cumulative data, we propose a model for aberrant cellular differentiation events in renal carcinogenesis that includes a loss of DTFs that controls renal differentiation and is followed by EMT and adipogenic transdifferentiation, which leads to ccRCC (**Fig. 8**).

## Discussion

Our study links biological pathways in ccRCC using gene array and pathway analyses of patient-matched normal renal and ccRCC tissues. Furthermore, we have validated a number of genes that are altered in ccRCC that were previously undefined. The ccRCC pathway signature is largely characterized by three major biological alterations: 1) *Loss of renal function* that is reflected by down-regulation of pathways related to normal renal function, such as excretion and electrolyte and ion channels. It is likely linked to a loss of renal epithelial differentiation since the pathway-related renal functions are performed by renal epithelial cells. 2) *Down-regulation of various metabolic pathways*, which indicates a significant metabolic alteration in ccRCC compared to normal renal cells. The metabolic pathways at the top of our pathway list include butanoate, propanoate, and valine, leucine and isoleucine degradation. These were also down-regulated in another study [29], in which analysis of ccRCC was performed using proteomics and metabolic profile. These pathways are also down-regulated in metastatic colon cancer [30]. Some of these metabolic changes are likely related to VHL gene mutation and/or the Warburg effect [31], [32]. 3) The *activation of immune pathways*, especially antigen presenting and processing pathways, is quite striking. The same finding is seen when ccRCC is compared to papillary RCC (data not shown). In addition, an overexpression of immune genes has been previously reported [33]. This immune gene expression signal is likely coming from tumor infiltrating immune cells; however, aberrant immune gene expression by ccRCC cells is also contributory, shown by our elevated TLR2 and CXCR4 expression data. Increased expression of both of these proteins has been linked to progressive renal disease and tumor formation/angiogenesis [34];[35];[36]. It is common for ccRCC to contain areas of necrosis, especially when the tumor grows faster than the blood supply can deliver nutrients. TLR2 can interact with a number of damage-associated molecular patterns (DAMPs) that are generated in the wake of cell death [37];[38];[39]. TLR2:DAMP interactions are important in the generation and maintenance of chronic inflammation that is integral to the disease process. Similarly, the homeostatic chemokine stromal cell-derived factor-1 (CXCL12/SDF-1) regulates many vital biological processes, including neovascularization and tumorigenesis via binding to the widely expressed receptor CXCR4 [40]. CXCR4-expressing tumor cells preferentially metastasize to tissues that highly express CXCL12, including the lung, liver, LN, and bone marrow [41];[42]. Interestingly, CXCR4 expression within a tumor increases, through HIF-1α, as the oxygen concentration decreases; this action enhances the metastatic potential of the tumor cells [43]. Consequently, there is a great deal of interest in developing anti-cancer therapeutics targeting CXCR4 [44]; [45]. It has been previously shown that other immune genes such as TLR3 [46] and B7-H1 ligand [47], are aberrantly expressed in ccRCC cells. The activated immune state may be linked to the responsiveness of ccRCC to immunotherapies, which makes ccRCC one of only a few cancers that responds to immune therapies [48]; [49].

Our data indicate that ccRCC has undergone EMT with an acquisition of mesenchymal/adipogenic differentiation signature and a loss of epithelial differentiation. Our work confirms a recent publication that uses 177 ccRCC samples and demonstrates a wound-healing signature in ccRCC that the authors associated with EMT [50]. The authors concluded that concomitant loss of differentiation and acquisition of a wound-healing signature was associated with EMT. Moreover, this signature was highly prognostic for poor survival.

We have revealed a gene expression signature and IHC findings that are consistent with adipogenesis in ccRCC. Moreover, our differentiation experiments have validated that ccRCC cells can undergo adipogenic transdifferentiation. It is well known that clear cell morphology is quickly lost when ccRCC cells are grown in standard cell culture media. In adipogenic media, ccRCC cells become adipogenic and redevelop clear cell morphology accumulating lipids, recapitulating the *in vivo* phenotype. It is interesting that *in vitro* adipogenic differentiation also leads to glycogen accumulation, which is another biological event responsible for clear cell morphology. These findings indicate that the clear cell morphology is a phenotypic marker of EMT, since adipogenic differentiation is a mesenchymal event.

Our *in vitro* differentiation experiments reveal a pluripotent mesenchymal stem cell-like quality in ccRCC cells with the capacity to undergo adipogenic and osteogenic transdifferentiation. This stem cell-like quality belongs to the tumor cell population but not to the patient-matched normal renal epithelial cell population. Previous studies show that cells induced to undergo EMT can acquire stem cell-like qualities [51]. We believe that the stem cell-like qualities observed in ccRCC are directly linked to EMT. Because ccRCC cells are capable of pluripotent differentiation *in vitro* and undergo adipogenic differentiation preferentially *in vivo* in human patients, it is likely that the adipogenic process is an important component of renal carcinogenesis. It is also possible that preferential adipogenic differentiation may be due to prevailing ecological factors in the kidney. Heuristically, we think that cellular differentiation experiments using differentiation media is useful for determining mesenchymal stem cell-like qualities in cancer cells. In addition, the stem cell-like characteristic of ccRCC may be responsible for its notorious drug resistance [52]; [53]. Recently, active compounds, such as Salinomycin, with cytotoxicity against stem cells were identified and used in cells with induced EMT changes and stem cell-like features [54].

Pathway and cellular differentiation changes in ccRCC may be partly mediated by a loss of four developmental transcription factors identified in our study. Since transcription factors typically regulate multiple genes, dramatic attenuation of an expression of four DTFs could have a major impact on biological function in ccRCC. One of the DTFs, GATA3, may play a crucial role in ccRCC, as it can potentially regulate all three biologic alterations identified in ccRCC: loss of renal epithelial differentiation, adipogenic differentiation, and immune activation. *GATA3* is important for renal development [55]; [56] and is necessary for nephric duct morphogenesis in the pro/mesonephric kidney [57]. A novel mutation in the GATA3 gene that results in haplodeficiency causes HDR syndrome (hypoparathyroidism, deafness, renal dysplasia) [58]; [59]. GATA3 also inhibits adipogenesis [20]; [21] and regulates immune function [60]. Because TFCP2L1 controlled genes are down-regulated in ccRCC and it is known to mediate renal epithelial cell development and tubule formation, loss of TFCP2L1 may play an important role in epithelial de-differentiation. *TFAP2B* is a member of the AP-2 family of transcription factors that functions as both a transcriptional activator and repressor. It appears to be required for proper terminal differentiation and function of renal tubular epithelia [61] and is integral to the survival of renal epithelial cells during renal development [62]. *DMRT2* is the top-ranking gene in our ccRCC gene expression dataset with the highest discriminant power based on our analysis using Fisher discriminant analysis (FDA) (Data not shown). This transcription factor is important for myogenesis and sex determination [63]. DMRT2 is also expressed in the urogenital tract [64] and its mutations are associated with renal abnormalities [65].

Based on our findings, we propose a model (**Fig. 8**) for aberrant cellular differentiation in ccRCC. Developmentally, kidneys are mesenchymal in origin and develop by biological processes, which includes mesenchymal epithelial transition (MET) [66]. We believe this process is reversed in ccRCC, which results in EMT and dedifferentiation. In our model, normal renal epithelial cells experience dedifferentiation and EMT as well as preferential adipogenic differentiation to develop an adipogenic phenotype in ccRCC. As a result of EMT and dedifferentiation, we postulate that ccRCC cells possess stem cell-like qualities. Since our samples came from patients with early-stage ccRCC, it appears that EMT plays a prominent role in renal carcinogenesis. This finding is in line with the important role of EMT pathogenesis of cancer [67].

Our present findings shed new light on the biology of ccRCC and have implications for future research. We showed a comprehensive pathway signature and identified an adipogenic transdifferentiation signature in ccRCC. We revealed that clear cell morphology is a marker of adipogenic transdifferentiation and EMT. We identified four DTFs, which may play a role in ccRCC due to loss of expression. Furthermore, our *in vitro* differentiation experiments may be useful as an assay for determining the pluropotency of cancer cells that have undergone EMT. With an implication for cell models used in ccRCC research, we showed that adipogenic media can restore the clear cell morphology in ccRCC cell lines. Since these cells exhibit the characteristic ccRCC phenotype, we suggest that they may be another relevant in vitro model to study ccRCC.

## Materials and Methods

This study was approved by the Mayo Institutional Review Board Committee. The tissues used in this study were either de-identified or were archival tissues and thus patient consent is not necessary.

### Study Samples

The tissue samples for the microarray study consisted of 10 patient-matched normal renal cortex and ccRCC tissues, five from stage I and five from stage II ccRCC. Both early-stage I and II tumors were localized disease; stage I tumors were less than 7 cm and stage II tumors were greater than 7 cm. We saw little to no difference in gene expression between the two tumor stages. Validation by real time quantitative PCR (qPCR) was performed using an independent set of 15 early-stage ccRCC patient-matched normal and renal samples. Another independent set of patient samples was used to measure protein levels by immunohistochemistry (IHC) in tissue arrays comprised of 50 paired early-stage ccRCC patient-matched normal and renal samples for immunohistochemical (IHC) validation. An *n* value was assigned for each IHC that indicated scorable samples from the tissue arrays. Rarely were all array samples intact, which allowed all 50 samples to be read. The pathologic diagnosis was confirmed by a central pathology review and was approved by the institutional review board.

### Microarray Protocols

DNA microarray experiments were performed using the Affymetrix HG-U133 set, which consisted of HG-U133a and HG-U133b gene arrays (Affymetrix, Santa Clara, CA) in accordance with standard protocols. The U-133 set included almost 45,000 probe sets, which included approximately 33,000 well-substantiated human genes. The background correction, probe summarization, and data normalization was performed with the GCOS 1.2 (Affymetrix, Santa Clara, CA) that used Affymetrix default analysis settings and global scaling as a normalizing method. Gene expression data from the two chips were combined with a scaling methodology. The gene expression data and the details of the data processing and methodology were deposited at Gene Expression Omnibus (Accession#GSE-6344). To combine HG-U133 set A and B chip data we used the 100 normalizing probe sets, which scaled the set B to set A and created a combo chip with 44760 probe sets. After the combo chip was made we used “trimmed mean” to scale the chips and filter out the genes with detection call “Absent in ALL” the chips and absolute fold change less than 2 in all of the possible pairwise comparisons. This resulted in 27,763 probe sets. We performed ANOVA on this probe set to determine statistical significance. Probes with p-value<0.05 totaled 13,729 genes. We used this gene set for our final analysis.

### Bioinformatics methods

#### Pathway analysis

Pathway analysis using the SigPathway package has been described elsewhere [68]. The SigPathway package [8] version 1.1.3 is available as a Bioconductor package (http://www.bioconductor.org) and employs two statistical parameters (NTK and NEK) to rank the pathways. NTK is a measure of the degree that a given pathway differs from the other pathways. NEK is a measure of the degree that the pathway composite expression differs between phenotypes. Pathways were ranked according to the average of the rank-orders of NTK and NEK; false discovery rate (q value) was calculated to adjust for multiple testing. The rank must be high in both rank-orders to minimize false positives.

#### Cluster analysis

Cluster 3.0 [69] was used to perform hierarchical cluster analyses using uncentered correlation and average linkage. Cluster analyses were performed on the most differentially expressed genes that were related to three pathway categories: 1) greater than two-fold difference for renal function-related genes (**Fig. 1a**), 2) greater than seven-fold difference for metabolic genes (**Fig. 1b**) and 3) greater than four-fold difference for immune genes (**Fig. 1c**); the statistical cutoff was P<0.05. Hierarchical cluster analyses were also performed on real time qPCR data on validated genes (**Fig. 2a**), adipogenesis genes (**Fig. 4a**), and EMT genes (**Fig. 7a**). Genetree methodology in GeneSpring (version 7.3, Agilent Technologies, Santa Clara, CA) was applied for cluster analysis that used Pearson correlation and average linkage on four DTFs (**Fig. 3a**), TFCP2L1, and its regulated genes (**Fig. 3c**). Fold changes and statistical significance of genes by analysis of variance (ANOVA) were performed in GeneSpring (version 7.3, Agilent Technologies, Santa Clara, CA). Multiple testing correction was done by the Benjamini and Hochberg method.

We analyzed an independent ccRCC gene expression database (GSE#15641) which was deposited at Gene Expression Omnibus (GEO) to validate our adipogenic and EMT signatures. The data was generated using gene array HG-U133A and preprocessed using MAS5 (Affymetrix, Santa Clara, Ca). We analyzed stage 1 and 2 ccRCC (n = 13) and normal samples (n = 23). Heatmaps were derived using our adipogenic and EMT signature gene lists (**Fig. S2 and S3**) through hierarchical clustering with uncentered correlation metric and average linkage method (Cluster 3.0).

### RNA isolation and quantitative PCR

Real time PCR (qPCR) was used to measure the changes in the mRNA levels of multiple genes from three different pathway categories in patient-matched renal samples. Total mRNA was isolated from tissue using Totally RNA (Ambion, Austin, TX), per the manufacturer's protocol, followed by ethanol precipitation and Chromaspin column. The O.D. 260/280 ratio of the mRNA was at least 1.8 and the 18S/28S bands were verified on a 1% agarose gel. The RT step was achieved by synthesizing cDNA from 3 µg RNA using the High Capacity Reverse Transcription kit as per the manufacturer's protocol (Applied Biosystems Foster City, CA). Applied Biosystems' assays-on-demand assay mix of primers and TaqMan® MGB probes (FAM™ dye-labeled) for the genes listed in **Table S1** were used for qPCR in a high throughput TILDA platform. Data were normalized to POLR2A for each sample and ΔCT values were calculated in matched normal and tumor patients. Samples were also normalized to UBC and KPNA6 for verification. Data were presented as a heatmap using cluster 3.0: uncentered correlation and incomplete linkage were used (**Fig. 2a**).

### Immunohistochemistry

IHC was performed on patient-matched renal samples to validate genes from three pathway categories at the protein level. Renal tissues were mounted on slides either from paraffin-embedded blocks for IHC or from frozen sections for Oil Red ‘O’ staining (Sigma-Aldrich, St. Louis, MO), per the manufacturer's protocol. Samples were blocked with Diluent that contained Background Reducing Components (Dakocytomation, Denmark) for 30 minutes and probed for specified antibodies that included: KNG1, AQP2, TFAP2B, ENO2 (Santa Cruz, Santa Cruz, CA), SCNN1B, TLR2, GATA2 (Novus, Littleton, CO), CXCR4 (BD Pharmingen, San Diego, CA), CYP2J2, ALDOB (Abcam, Cambridge, MA), DMRT2, TFCP2L1 (Lifespan Biosciences, Seattle, WA), Vimentin (Sigma-Aldrich), and ADFP (Acris Antibodies, Germany). Negative sections were prepared by incubating the slides in the absence of the primary antibody. The staining of the tissue sections was scored and verified by a pathologist (KW) based upon signal intensity (0-3+) and the percentage of positive cells (0≤5%, 1+ = 5-20%, 2+ = 20-50%, and 3+≥50%). Cases were excluded from the study if a section could not be assigned a score due to insufficient tumor tissue. Afterwards, the grading score was summed and reported as summed scores followed by Chi-square analysis (df = 3, p<0.01). Images were obtained at 20X using Scanscope XT and Imagescope software (Aperio Technologies, Vista, CA).

### Cell culture and differentiation experiments

The human A498 ccRCC cell line and MDCK canine normal renal cells were purchased from ATCC (Manassas, VA) while KIJ-265 (Stage 4) and KIJ-308 (Stage 2) cell lines and primary cells were established in the Copland laboratory and derived from human renal clear cell carcinoma and normal-matched tissues. Renal cells were maintained in complete renal media containing MEM (Cellgro Herndon, VA) supplemented with 10% fetal bovine serum (Hyclone Logan, UT), 1 mM sodium pyruvate, 10 mM HEPES, 1% non-essential amino acids and penicillin-streptomycin-amphotericin B (Cellgro Herndon, VA) at 37°C in a humidified atmosphere with 5% CO_2_. For differentiation experiments, cells were maintained for 2 weeks in complete renal media, adipogenic, or osteogenic media. For adipogenic differentiation, media was supplemented for 2 weeks with 0.1 µM dexamethasone, 10 µg/mL insulin, 100 µM indomethacin, and 500 µM isobutylmethylxanthine (Sigma-Aldrich, St. Louis, MO) followed by Oil Red ‘O’ and PASH stains, per the manufacturer's protocol (Richard Allan Scientific, Kalamazoo, MI). For osteogenic differentiation, media was supplemented for 2 weeks with 50 µM Ascorbic acid, 0.1 µM Dexamethasone, and 10 nM beta-Glycerolphosphate (Sigma-Aldrich, St. Louis, MO) and staining with Alizarin Red S, per the manufacturer's protocol (Sigma-Aldrich, St. Louis, MO).

## Supporting Information

Table S1 Table of primers used in TILDA qPCR. KPNA6, POLR2A and UBC were used as internal controls.
(0.06 MB DOC)

Figure S1 Renal developmental genes are down-regulated in ccRCC. The large heatmap shows differential expression of transcription factors between ccRCC and normal renal samples. The smaller heatmap highlights clustering of down-regulated renal developmental transcription factors (GATA3, TFAP2B, DMRT2, and TFCP2L1) in a subcluster. Upregulation of genes is indicated in red, downregulation is indicated in green, and a similar expression is indicated in black, as generated by Cluster 3.0.
(0.69 MB TIF)

Figure S2 An adipogenic gene expression signature of ccRCC is elucidated. Normal denotes normal renal samples. Gene expression data are from a publicly available database, GSE#15641, deposited at Gene Expression Omnibus (GEO). Details on the clustering method are mentioned in the text. Red color indicates an upregulation of genes, green indicates a downregulation, and black indicates a similar expression.
(0.49 MB TIF)

Figure S3 An epithelial mesenchymal transition (EMT) gene expression signature of ccRCC is identified. Normal denotes normal renal samples. Gene expression data are from a publicly available database, GSE#15641, deposited at Gene Expression Omnibus (GEO). Details on the clustering method are mentioned in the text. Red color indicates an upregulation of genes, green indicates a downregulation, and black indicates a similar expression.
(0.66 MB TIF)
