# Supplementary material for: Pathway Signature and Cellular Differentiation in Clear Cell Renal Cell Carcinoma
Source: PLoS One. 2010 May 18;5(5):e10696. doi: 10.1371/journal.pone.0010696 (PMC2872663; doi:10.1371/journal.pone.0010696)
Supplement: Table S1 — Table of primers used in TILDA qPCR. KPNA6, POLR2A and UBC were used as internal controls. (0.06 MB DOC) [file pone.0010696.s001.doc]

| **Primer** | **Assay** | **Primer** | **Assay** |
| --- | --- | --- | --- |
| ABAT | Hs00609436_m1 | INHBB | Hs00173582_m1 |
| ADM | Hs00181605_m1 | KCNJ1 | Hs00165012_m1 |
| ALDH4A1 | Hs00186689_m1 | KNG1 | Hs00949376_m1 |
| ALDOB | Hs00163626_m1 | KPNA6 | Hs00202389_m1 |
| ANGPT2 | Hs00169867_m1 | MAL/CD8A | Hs00233520_m1 |
| AQP2 | Hs00166640_m1 | MET | Hs00179845_m1 |
| ATP6V0A4 | Hs00220986_m1 | NELL1 | Hs00196243_m1 |
| BHLHB3 | Hs00229146_m1 | NPHS2 | Hs00387817_m1 |
| C1QA | Hs00381122_m1 | PAG1 | Hs00179693_m1 |
| C3 | Hs00163811_m1 | PAH | Hs00609359_m1 |
| CALB1 | Hs00191821_m1 | PCK1 | Hs00159918_m1 |
| CAV2 | Hs00184597_m1 | PDK1 | Hs00176853_m1 |
| CDH4 | Hs00242399_m1 | PLG | Hs00264877_m1 |
| CLCNKA | Hs00427895_g1 | PLK2 | Hs00198320_m1 |
| CLCNKB | Hs01114443_m1 | POLR2A | Hs00172187_m1 |
| CYP2J2 | Hs00356035_m1 | PSMB8 | Hs00544758_m1 |
| CYP4F2 | Hs00426608_m1 | RGS1 | Hs00175260_m1 |
| DMRT2 | Hs00246364_m1 | RGS5 | Hs00186212_m1 |
| EGLN3 | Hs00222966_m1 | SERPINH1 | Hs00241844_m1 |
| EHF | Hs00171917_m1 | SLC12A1 | Hs00165731_m1 |
| ENO2 | Hs00157360_m1 | SLC13A3 | Hs00224536_m1 |
| ESRRG | Hs00155006_m1 | TCF4 | Hs00162613_m1 |
| FABP7 | Hs00361426_m1 | TFAP2B | Hs00231468_m1 |
| FGF9 | Hs00181829_m1 | TFCP2L1 | Hs00232708_m1 |
| FLT1 | Hs00176573_m1 | TLR2 | Hs01014511_m1 |
| FREM1 | Hs00381549_m1 | TNFAIP6 | Hs00200180_m1 |
| GAS2L3 | Hs00604175_m1 | TREM2 | Hs00219132_m1 |
| HIG2 | Hs00203383_m1 | UBC | Hs00824723_m1 |
| HK2 | Hs00606086_m1 | VDR | Hs00172113_m1 |
| HLA-DQB1 | Hs03054971_m1 | VWF | Hs00169795_m1 |
| HPD | Hs00157976_m1 | WT1 | Hs01103749_m1 |
| HSD11B2 | Hs00388669_m1 |  |  |
