# Supplementary figures and images for: Pathway Signature and Cellular Differentiation in Clear Cell Renal Cell Carcinoma
Source: PLoS One. 2010 May 18;5(5):e10696. doi: 10.1371/journal.pone.0010696 (PMC2872663; doi:10.1371/journal.pone.0010696)

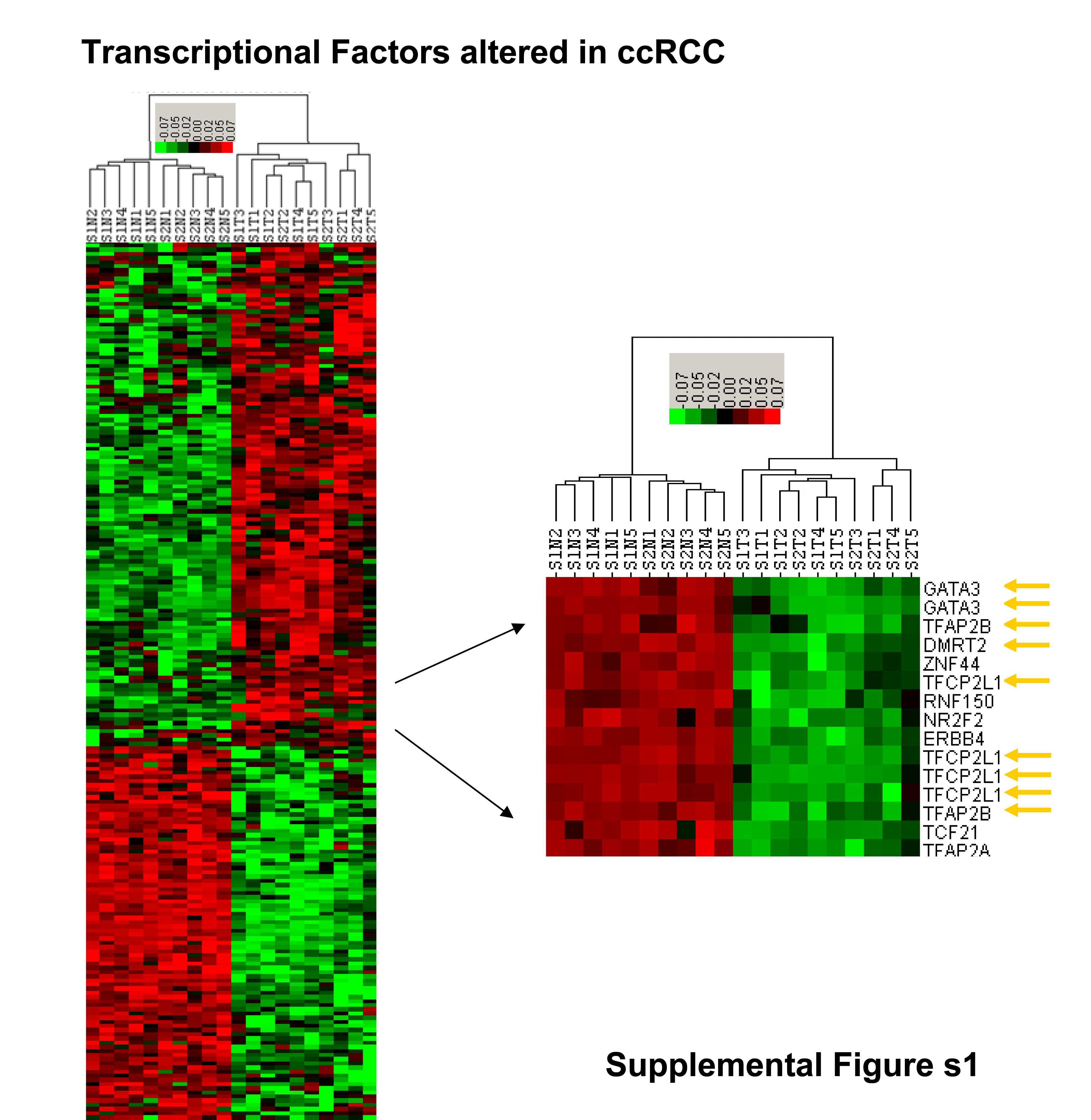

Supplement: Figure S1 — Renal developmental genes are down-regulated in ccRCC. The large heatmap shows differential expression of transcription factors between ccRCC and normal renal samples. The smaller heatmap highlights clustering of down-regulated renal developmental transcription factors (GATA3, TFAP2B, DMRT2, and TFCP2L1) in a subcluster. Upregulation of genes is indicated in red, downregulation is indicated in green, and a similar expression is indicated in black, as generated by Cluster 3.0. (0.69 MB TIF) [file pone.0010696.s002.tif]

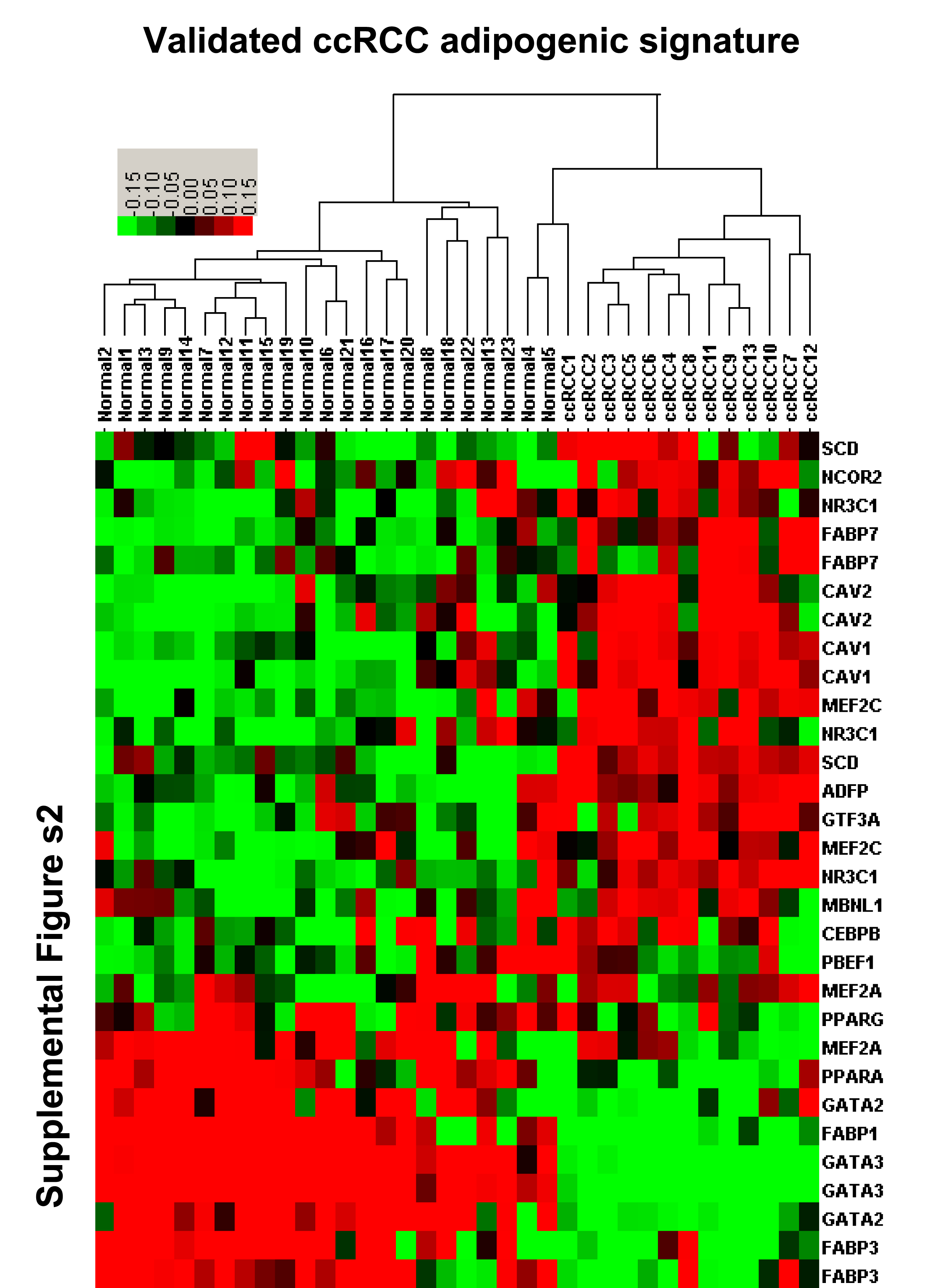

Supplement: Figure S2 — An adipogenic gene expression signature of ccRCC is elucidated. Normal denotes normal renal samples. Gene expression data are from a publicly available database, GSE#15641, deposited at Gene Expression Omnibus (GEO). Details on the clustering method are mentioned in the text. Red color indicates an upregulation of genes, green indicates a downregulation, and black indicates a similar expression. (0.49 MB TIF) [file pone.0010696.s003.tif]

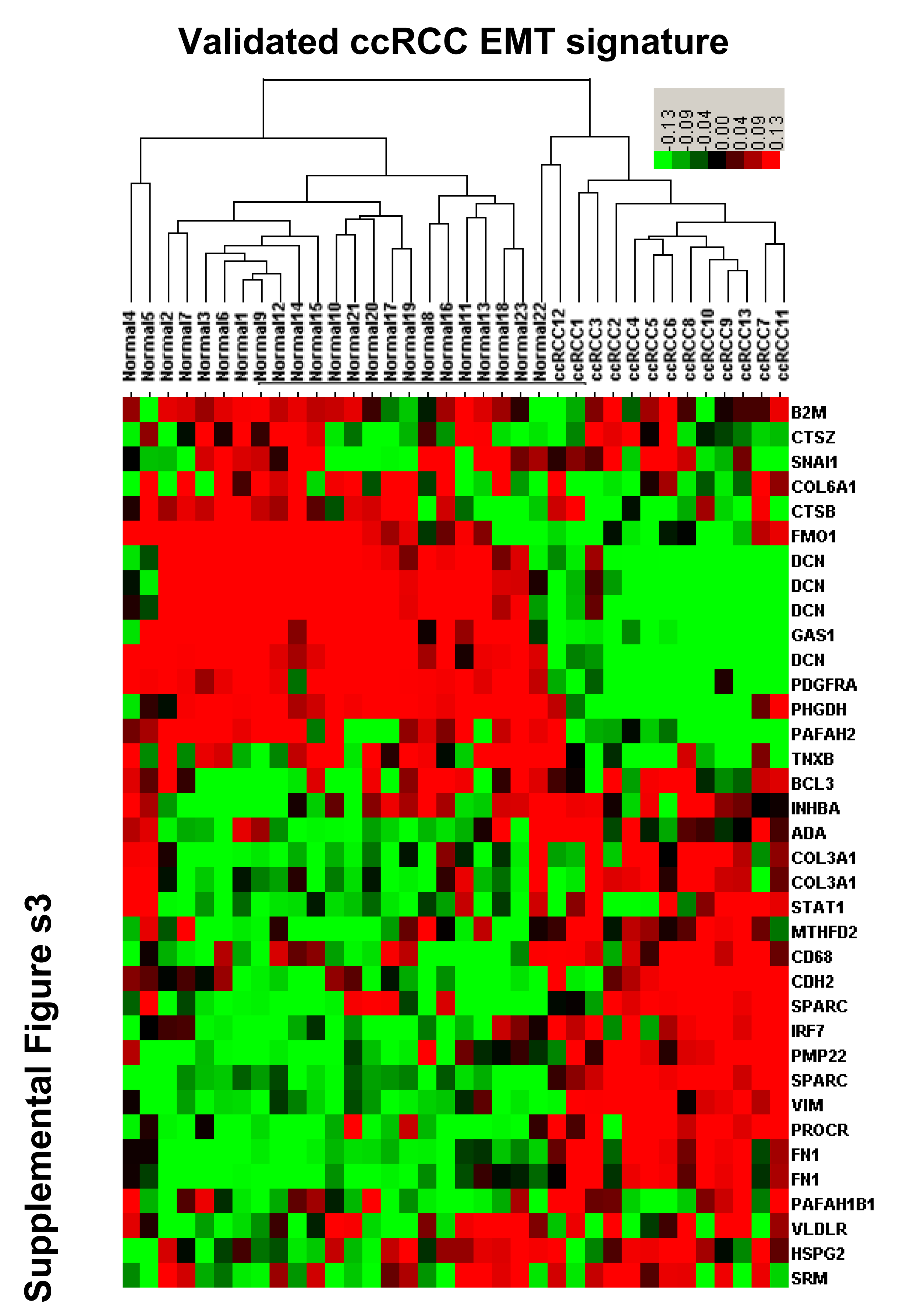

Supplement: Figure S3 — An epithelial mesenchymal transition (EMT) gene expression signature of ccRCC is identified. Normal denotes normal renal samples. Gene expression data are from a publicly available database, GSE#15641, deposited at Gene Expression Omnibus (GEO). Details on the clustering method are mentioned in the text. Red color indicates an upregulation of genes, green indicates a downregulation, and black indicates a similar expression. (0.66 MB TIF) [file pone.0010696.s004.tif]
